# Enhanced Oil Recovery Mechanism and Technical Boundary of Gel Foam Profile Control System for Heterogeneous Reservoirs in Changqing

**DOI:** 10.3390/gels8060371

**Published:** 2022-06-12

**Authors:** Liang-Liang Wang, Teng-Fei Wang, Jie-Xiang Wang, Hai-Tong Tian, Yi Chen, Wei Song

**Affiliations:** School of Petroleum Engineering, China University of Petroleum (East China), Qingdao 266580, China; llwang2017@163.com (L.-L.W.); wangjxupc@126.com (J.-X.W.); tian_haitong2022@163.com (H.-T.T.); chyi2020@163.com (Y.C.); song15954231226@163.com (W.S.)

**Keywords:** enhanced gel system, enhanced foam system, heterogeneous reservoir, plugging adaptability, enhanced oil recovery

## Abstract

The gel plugging and flooding system has a long history of being researched and applied, but the Changqing reservoir geological characteristics are complex, and the synergistic performance of the composite gel foam plugging system is not fully understood, resulting in poor field application. Additionally, the technique boundary chart of the heterogeneous reservoir plugging system has hardly appeared. In this work, reservoir models of porous, fracture, and pore-fracture were constructed, a composite gel foam plugging system was developed, and its static injection and dynamic profile control and oil displacement performance were evaluated. Finally, combined with the experimental studies, a technical boundary chart of plugging systems for heterogeneous reservoirs is proposed. The research results show that the adsorption effect of microspheres (WQ-100) on the surface of elastic gel particles-1 (PEG-1) is more potent than that of pre-crosslinked particle gel (PPG) and the deposition is mainly on the surface of PPG. The adsorption effect of PEG-1 on the surface of PPG is not apparent, primarily manifested as deposition stacking. The gel was synthesized with 0.2% hydrolyzed polyacrylamide (HPAM) + 0.2% organic chromium cross-linking agent, and the strength of enhanced gel with WQ-100 was higher than that of PEG-1 and PPG. The comprehensive value of WQ-100 reinforced foam is greater than that of PEG-1, and PPG reinforced foam, and the enhanced foam with gel has a thick liquid film and poor foaming effect. For the heterogeneous porous reservoir with the permeability of 5/100 mD, the enhanced foam with WQ-100 shows better performance in plugging control and flooding, and the recovery factor increases by 28.05%. The improved foam with gel enhances the fluid flow diversion ability and the recovery factor of fractured reservoirs with fracture widths of 50 μm and 180 μm increases by 29.41% and 24.39%, respectively. For pore-fractured reservoirs with a permeability of 52/167 mD, the PEG + WQ-100 microsphere and enhanced foam with WQ-100 systems show better plugging and recovering performance, and the recovery factor increases are 20.52% and 17.08%, 24.44%, and 21.43%, respectively. The smaller the particle size of the prefabricated gel, the more uniform the adsorption on the foam liquid film and the stronger the stability of the foam system. The plugging performance of the composite gel system is stronger than that of the enhanced gel with foam, but the oil displacement performance of the gel-enhanced foam is better than that of the composite gel system due to the “plug-flooding-integrated” feature of the foam. Combined with the plugging and flooding performance of each plugging system, a technique boundary chart for the plugging system was established for the coexisting porous, fracture, and pore-fracture heterogeneous reservoirs in Changqing Oilfield.

## 1. Introduction

Due to the stimulation of low/ultra-low permeability reservoirs, high-permeability channeling channels are easily formed after artificial fractures are connected with natural fractures [1,2]. However, the high cost of fine water injection and the long shutdown period greatly affect the normal production of oilfields [3]. Given this, the development of plugging and flooding systems with strong applicability has received extensive attention.

Gel and foam systems, as the two most widely used plugging agents, have achieved positive progress in both laboratory experiments and field applications in recent decades [4,5]. Gel-based plugging systems have been used on a large scale in the 1990s, and various modified gels, micro-nano microspheres, polyethylene glycol gels (PEG), and chitosan-based gels have been developed, including preformed particle gel (PPG) and HPAM weak gels with various particle sizes and compositions [6,7,8,9,10].

Field tests show that a single plugging system cannot meet the selective plugging of highly heterogeneous reservoirs and cannot meet the needs of adjusting the water absorption profile and enhancing oil displacement. In recent years, scholars have carried out a series of composite plugging system applicability evaluation experiments to achieve the comprehensive effect of “deep control and flooding + near-well plugging”. Jia et al., developed a novel nanocomposite gel with tunable gel formation time based on the in-situ polymerization method [11]. The nanocomposite gel system has good temperature resistance (75–105 °C), adding 5% nano-silica, and the compressive performance of the composite gel system is improved from 8.7 to 21 KPa; ammonium persulfate and hydrochloric acid can be caused to be effectively degraded. Given the development of cross-linking after polymer flooding, Haung et al., proposed a low initial viscosity gel plugging agent: 500–1000 mg/L polymer LH2500, 1000–2500 mg/L cross-linking agent CYJL, 200–500 mg /L citric acid, 100–150 mg/L sodium sulfite, and 100–200 mg/L sodium polyphosphate. For secondary polymer flooding reservoirs, the oil recovery can be enhanced by 13.6% after 0.1 PV gel plugging [12]. Liu et al., developed a gel system formed by a terpolymer (L-1) and a new cross-linking system (HB-1) for ultra-deep and high-temperature reservoirs [13]. This can create a stable continuous 3D network structure with good long-term thermal stability. The strength of the gel system can be adjusted by changing the concentrations of the terpolymer (0.05–1%) and the crosslinking agent (0.05–1%).

The commonly used foam sealing and channeling systems are mainly composed of a foaming agent, foam stabilizer, and gas phase [14,15]. Nitrogen comes from various sources and has become the most commonly used foam gas phase. Foam stabilizers include HPAM, worm-type surfactants, inorganic nanoparticles, and nano-microspheres. Using three crude oils with different properties, Lai et al., studied the stability and oil displacement capacity of the gel foam system [16]. For crude oil with more heavy components such as resins and asphaltenes, when the oil saturation is lower than 20%, the gel foam shows higher foam stability. The oil-containing gel foam generated from heavy oil has good stability, the plugging rate is 95.33%, and the enhanced oil recovery is 23.1%. Zhang et al., performed huff and puff experiments on core samples using different gases and foams at the temperature of unconventional liquid reservoirs, capturing time-lapse CT images with a computed tomography unit (CTU) [17]. Studies have shown that a combination of EOR techniques (foam or sequencing surfactant and gas injection) opens the possibility of achieving sound oil recovery. Natural fractures can easily lead to lost circulation and can easily limit the normal production of oil fields. Li et al., developed an oil-based pressure-bearing foam gel plugging agent for fracturing shale: 0.6% DSFA foaming agent + 0.3% BPMO foaming agent + 0.5% EPDM + 1.5% SBS + 0.05% rigid crosslinking agent DB + 0.02% modified SiO_2_ nanoparticles [18]. The core plugging experiment shows that the plugging efficiency of the plugging agent is about 90%.

The concise literature shows that many studies have been carried out on the plugging control and flooding system and its mechanism in heterogeneous reservoirs, and good application results have been achieved in oilfields. However, in the face of complex and changeable reservoirs with strong heterogeneity, coupled with insufficient understanding of the synergistic mechanism of multi-component compound plugging systems, the existing plugging systems have poor water blocking and oil-increasing effects, and even cause secondary damage to the reservoir. In addition, the technical chart of the heterogeneous reservoir plugging system has hardly appeared. Therefore, in this work, combined with the geological characteristics of Changqing Oilfield, reservoir models of porous, fracture, and pore-fracture were constructed, the composite plugging system was developed based on the gel and foam system, and injection and dynamic performance were evaluated. The synergistic effect stimulates the new vitality of traditional plugging agents, optimizes and strengthens the gel and gel-enhanced foam plugging control and flooding systems, and achieves breakthroughs in the channeling channel of “injecting, plugging, long-lasting, and low-cost”. Finally, based on the optimal plugging systems of different reservoir models, a technical boundary chart of plugging systems for heterogeneous reservoirs is proposed. The results of this study improve the composite gel foam plugging system and its action mechanism suitable for highly heterogeneous reservoirs; additionally, they provide crucial technical guidance for Changqing’s low-permeability reservoirs to achieve stable production and increase production.

## 2. Experiments and Methods

### 2.1. Static Performance Evaluation Experiment of Plugging System

#### 2.1.1. Gel Plugging Agent

1. Pre-crosslinking gel particle

The prefabricated gel particles include microspheres (WQ-100), PEG-1, PPG, HPAM, organochromium crosslinking agent, foaming agent, and formation water (60,000–100,000 mg/L), all provided by Changqing Oilfield. Microscopic tests of WQ-100, PEG-1, and PPG by Microscope First. A total of 0.5 g of prefabricated gel particle plugging agent and 100 mL of formation water/kerosene was sequentially added to 6 beakers and stirred with a magnetic stirrer for 10 min. Next, the magnetron was taken out and all the beakers were sealed and placed in a hot air drying oven with a constant temperature of 70 °C [19]. Finally, the microscopic morphology of WQ-100, PEG-1 and bulk particles was observed by the microscope, and the particle size changes were analyzed and counted.

2. Gel

A total of 500 mL of formation water was poured into a jar and stirred with a large torque stirrer. Slowly, 3 g of HPAM powder was added and was continued to be stirred until the HPAM was completely dissolved. The prepared mother liquor was diluted with formation water and stirred for 1 min to make HPAM evenly dispersed in the formation water. Next, 30 mL of the diluted HPAM solution was added to the cup, the organic chromium cross-linking agent was dropped into the beaker and stirred for 30 s (450 rpm), and 25 mL was poured into a stoppered measuring cylinder. All stoppered graduated cylinders were placed in a water bath preheated to 70 °C, the state of the mixture in the graduated cylinder was recorded every 1 h, and the time required for each group of formulations to reach grade G was recorded [20]. After the gel in the graduated cylinder reached the G level, the vacuum pump hose was connected to the inside of the forming gel, the vacuum pump was started, and the maximum reading of the vacuum gauge was recorded.

#### 2.1.2. Foam

A 100 mL quantity of distilled water was put into the mud cup and different volume fractions of foaming agents were added. The high-speed stirrer was set to 6000 rpm and the foam was poured out after stirring for 3 min. The foaming volume and the time it took to produce 50 mL of liquid were recorded. The foam performance is quantitatively evaluated using the foam comprehensive value, and the calculation method is as follows [21]:

$$F_{c}=V_{0}t_{f}$$

where $F_{c}$ is foam comprehensive coefficient, mL·s; $V_{0}$ is initial foam volume, mL; $t_{f}$ is foam half-life, s.

#### 2.1.3. Binary/Ternary Complex System

1. Pre-crosslinking gel particles

The formula of the pre-crosslinking gel particle complex systems were 0.5% WQ-100 + 0.5% PEG-1, 0.5% WQ-100 + 0.5% PPG, and 0.5% PEG-1 + 0.5% PPG, respectively. The formation water was used as the dispersant for the compound system, totaling 100 mL. After the three groups of compound systems were sealed, they were aged in a constant temperature oven at 70 °C for 12 h. The pre-gel compound system was taken out and the poorly dispersed system was uniformly dispersed again by stirring with a glass rod.

2. Enhanced gels with pre-crosslinking gel particle

The HPAN dispersion method is the same as 2.1.1 (2). All dispersion systems were put into 25 mL graduated cylinders with a stopper and placed in a water bath with a constant temperature of 70 °C, and the gel formation time and the degree of vacuum breakthrough were recorded.

3. Enhanced foam with pre-crosslinking gel particles and gels

The formulation and interfacial tension of the gel-reinforced foam compound system is shown in Table 1. A 100 mL quantity of prefabricated gel particle-foaming agent dispersion system was put into a mud cup. After stirring for 3 min, the rotation was stopped and immediately poured into a graduated cylinder to measure the foaming volume and absorb the upper foam for microscopic observation. The time when the liquid in the graduated cylinder reaches 50 mL was recorded and the comprehensive foam value of the system was calculated. The 0.2% HPAM + 0.2% organic chromium cross-linking agent + 0.4% foaming agent was dispersed in formation water to obtain a jelly-reinforced foam system. The reinforced foam system sample was added to the sample cell of the interfacial tension tester, and the size of the bubbles at the outlet of the injector in the tester was controlled by software. The test was stopped when each sample is measured for 900 s, and the real-time gas–liquid interfacial tension values in the interval of 300–900 s were averaged as the test results of this group of samples [22].

### 2.2. Physical Simulation Model Construction

**Figure 1:**
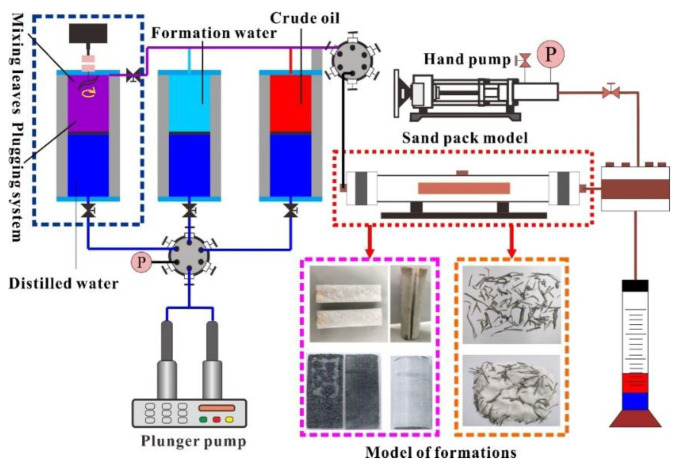
Flow chart of laboratory displacement experiment.

To better simulate the reservoir conditions of strong heterogeneity with the development of micro-fractures, laboratory physical simulation experimental models of porous, fracture, and pore-fracture were constructed. For the porous reservoirs model, a one-dimensional sand filling model was filled by mixing quartz sands of different particle sizes, with a porosity of 26% and a permeability of 7 × 10^−3^ μm^2^. Secondly, the artificial core (5 × 10^−3^ μm^2^) was split along the axis direction by the Brazilian splitting device, and the split core adhered to the ceramsite. After the cores were merged, they were wrapped and tightened by the raw material belt to simulate the fractured reservoir after underground fracturing, and the opening of the fracture could be changed by adjusting the particle size of the ceramsite. Finally, the sand filter screen was cut into small strips and mixed with quartz sand (40–80 μm, 80–120 μm) evenly. The one-dimensional sand-packing model was filled to construct pores for simulating reservoirs with micro-fractures. The diagram and fundamental porosity and permeability characteristics of the fractured and pore-fractured experimental models are shown in Figure 1 and Table 2, respectively.

The calculation formula of fracture permeability is as follows.

$$K=1000\times \frac{\phi b^{2}}{12}$$

where K is permeability, 10^−3^ μm^2^, ϕ is porosity of fractured reservoir model, and b is fracture width, μm.

### 2.3. Plugging EOR Mechanisms and Performance

The prefabricated different reservoir models were loaded into the displacement system, and the experimental flow of plugging and enhanced oil recovery displacement is shown in Figure 1. The formation water was pumped into the model to measure the initial permeability (Ki), the fracture opening was adjusted by changing the particle size of ceramsite, and the number of screen bars was increased or decreased to alter the pore-fractured reservoir model. For each reservoir model, first, a plunger pump was used to saturate the model with water at 0.5 mL/min and record the steady pressure Pi. The injection rate was maintained and 0.5 PV of the plugging system was injected into the model to be tested. After the plugging system was injected, it was transferred to the formation water injection until the pressure was stable.

It is worth noting that for millimeter-centimeter PPG, it can be seen from the static performance experiments that it has evident stratification after adding liquid. During the injection and displacement process, the conventional intermediate container was used because a large amount of PPG is deposited or adheres to the top cover of the intermediate container. This can cause injection difficulties and even lead to the experiment’s failure. As shown in Figure 1, the intermediate vessel injected with PPG was upgraded and improved in this study, mild shear was generated by stirring, and the pressure-resistant pipeline was thickened with an inner diameter of 3 mm. The slightly sheared bulk particles are further subjected to extrusion shearing while passing through the injection pipeline, which also improves the PPG injection performance. During the EOR performance evaluation experiment, after saturating the formation water and crude oil in sequence, firstly, the formation water was injected to stimulate the production. The produced fluid was injected into the plugging system after no oil phase flowed out, and then the formation water was injected again until no oil phase flowed out again. During the period, the cumulative oil production was recorded by the oil-water separation metering device, the cumulative oil production injection curve was drawn, and the enhanced oil recovery performance of the plugging system was evaluated. The plugging rate represents the plugging ability of the formation after a particular plugging agent is injected and forms stable plugging. Its calculation theory is as follows:

$$\mathrm{Plugging}\;\mathrm{efficiency}=1-\frac{K_{1}}{K_{i}}$$

where $K_{i}$ is permeability after plugging, 10^−3^ μm^2^, $K_{1}$ is initial permeability, 10^−3^ μm^2^.

## 3. Results and Discussion

### 3.1. Static Performance of Plugging System

#### 3.1.1. Gel Plugging Agent

1. Pre-crosslinking gel particle

**Figure 2:**
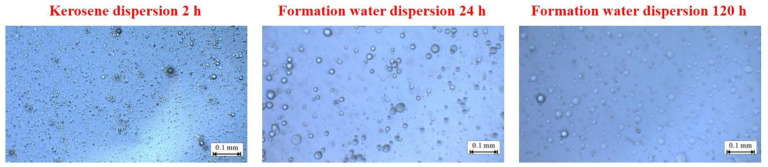
Micrograph of microspheres (WQ-100) after dispersion.

Figure 2 shows the particle size test results of the microspheres (WQ-100) dispersed in the formation water and kerosene. WQ-100 delivers good dispersing performance in both formation water and kerosene, has good adaptability to both dispersing media, and has no precipitation occurs. WQ-100 still showed good expansion performance under high temperature and high salt environments, and the particle size distribution expanded from 1–5 to 10–20 μm within 24 h. Additionally, WQ-100 has good stability without swelling, cracking, and cross-linking polymerization within 120 h.

**Figure 3:**
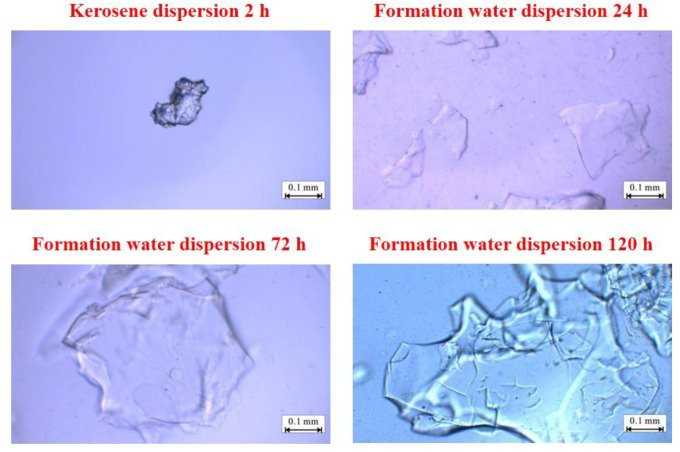
Micrograph of PEG-1 after dispersion.

Figure 3 shows the particle size test results after PEG-1 formation water and kerosene are dispersed. When sampling the PEG-1 dispersion system, delamination can be observed, indicating that the dispersibility of the plugging agent in formation water is not excellent. PEG-1 continued to expand within 120 h and the expansion rate gradually decreased with time. The particle size was raised from 0.1 to 0.5 mm and the diameter expansion rate was 500%. The PEG-1 gel particles had good temperature and salt resistance. During the ageing process, the PEG-1 particles continued to absorb water, and the inner folded network of long chains gradually unfolded. At the macroscopic level, the particle volume expanded, the surface gradually became smooth, and the interior of the particles became transparent.

**Figure 4:**
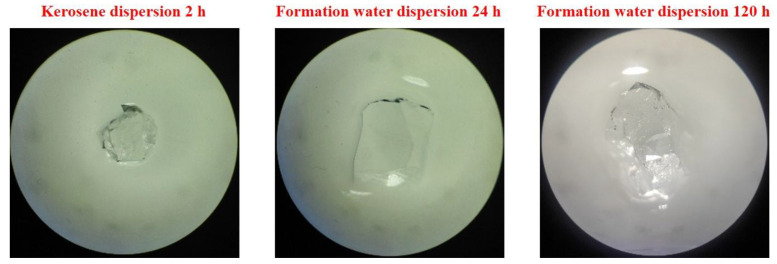
Micrograph of preformed particle gel after dispersion.

The particle size test results of bulky particles dispersed in formation water and kerosene are shown in Figure 4. When sampling the bulky particle dispersion system, an apparent layering phenomenon was found, and reliable sampling could be carried out only after stirring. The swelling particle samples continued to expand within 120 h, the expansion rate decreased with time, and the particle size grew from 5 to 10 mm.

2. Gel

**Figure 5:**
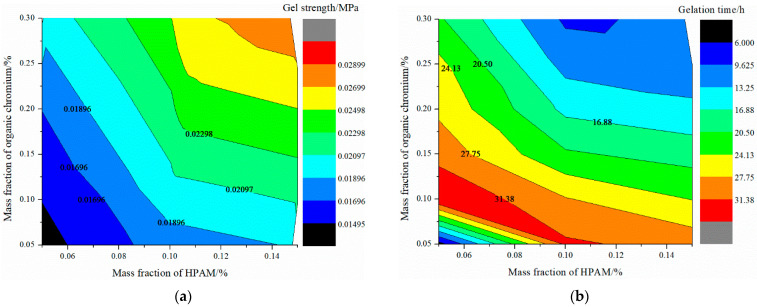
Contour diagrams of static performance of organic chromium gel: (**a**) gel strength, (**b**) gelation time.

After HPAM was dispersed, it was a uniform viscous colloidal fluid. No residue was seen at the bottom of the bottle, indicating that it had good adaptability to the formation water with high salinity. The breakthrough vacuum degree and gel formation time of the gel is shown in Figure 5. With the increase of the mass fraction of HPAM and organic chromium cross-linking agent, the gelation time of the gel was shortened, and the strength of the gel was increased. When the mass fractions of HPAM and organic chromium crosslinking agent are 0.05–0.15% and 0.05–0.25%, respectively, the gelation time is 11–35 h and the gel strength is 0.015–0.027 MPa.

**Figure 6:**
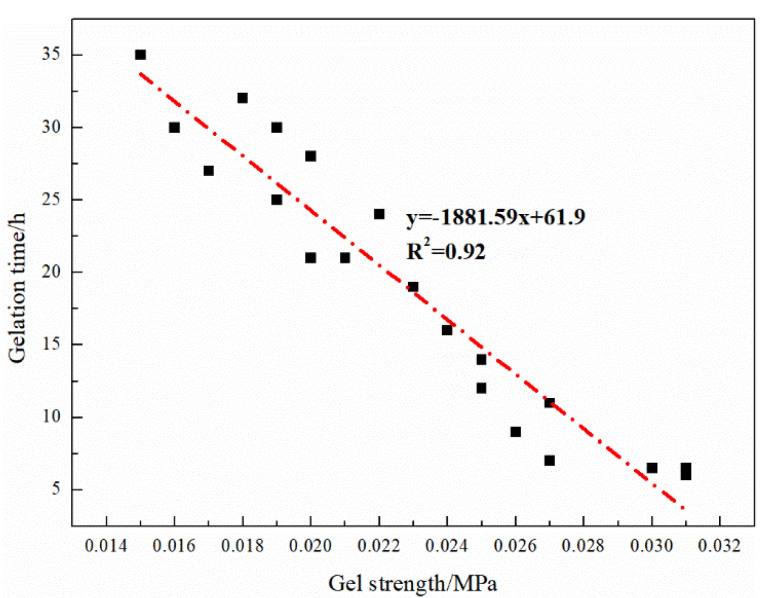
Fitting curves of breakthrough vacuum and gelation time.

The formulation adjustment of the gel system is more flexible. The concentration of each component can be adjusted according to the need to change the gelation time and the breakthrough vacuum degree to achieve the effect of deep displacement regulation and enhanced plugging. There is an obvious negative correlation between the breakthrough vacuum degree of the gel system and the gelation time, as shown in Figure 6. In order to meet the fluidity and blocking performance, the recommended gel system formulation is 0.2% HPAM + 0.2% organic chromium cross-linking agent.

#### 3.1.2. Foam Comprehensive Coefficient at Varying Concentrations of Foaming Agent

**Figure 7:**
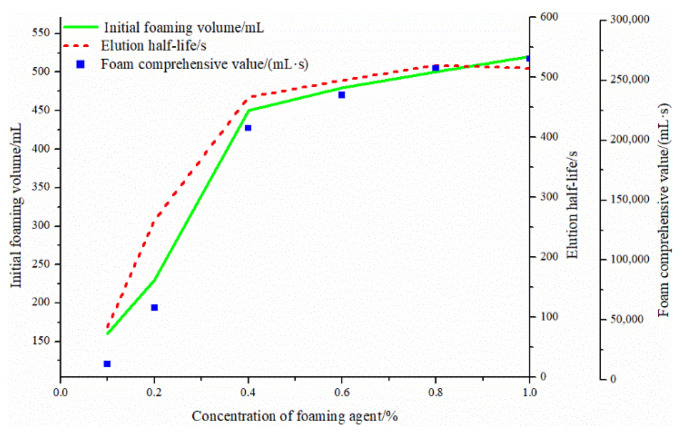
Concentration of foaming agent-initial foaming volume, foam half-life, and composite value.

As a surfactant, a foaming agent can be evenly distributed on the gas–liquid interface and slow down the time required for gas–liquid separation. The foaming performance and half-life results of different concentrations of foaming agents are shown in Table 3 and Figure 7. As the concentration of foaming agent (c) increased from 0.1% to 0.4%, the initial foaming volume increased from 160 mL to 450 mL. At c > 0.4%, the initial bubble volume and elution half-life changed gradually, and after c > 0.8%, the elution half-life decreased. With the increase of the foaming agent concentration, the density of the surfactant on the liquid film gradually increases after the foam is formed. After the monomolecular film is completely adsorbed on the surface of the liquid phase, the bimolecular film begins to form. The concentration of the foaming agent is defined as the critical concentration of the foaming agent. Above this concentration, the comprehensive performance of the foam begins to stabilize.

#### 3.1.3. Binary/Ternary Composite Plugging System

1. Pre-crosslinking gel particle

**Figure 8:**
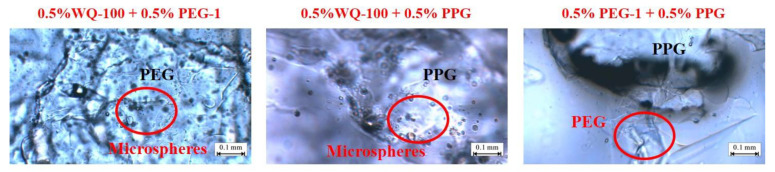
Pre-crosslinking gel particle absorptive effect.

The microscopic test results of the gel particle compound system are shown in Figure 8. In the compound system of WQ-100 and PEG-1/PPG, the primary performance is adsorption. The adsorption effect of WQ-100 on the surface of PEG-1 particles is stronger than that on the surface of PPG particles. It is worth noting that the adsorption amount of WQ-100 on the PPG surface is much less than the amount deposited on the surface recesses, which indicates that the microspheres are mainly deposited on the surface of PPG particles. In addition, due to the large particle size of PEG-1 and PPG particles, the adsorption effect of PEG-1 on the surface of PPG particles is not obvious, mainly manifested as deposition stacking. WQ-100 can form secondary plugging on the formation after PEG plugging, improving the sweeping flow efficiency.

2. Enhanced gels with pre-crosslinking gel particle

**Figure 9:**
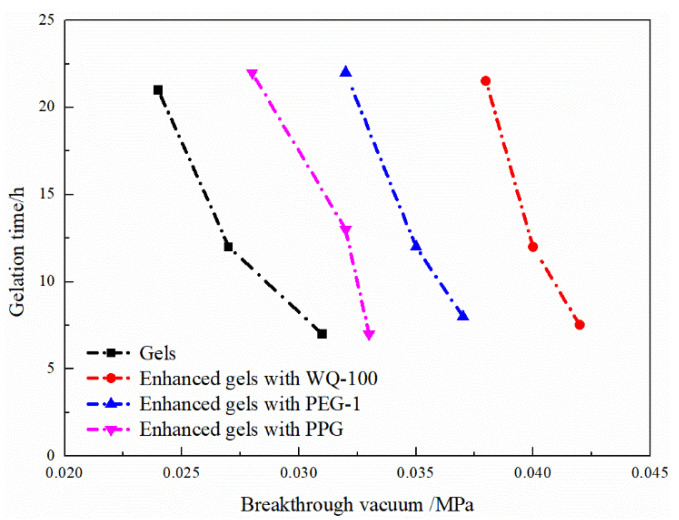
Gels strength and gelation of enhanced gels systems.

The gelation time and strength of the prefabricated gel particles and the polymer gel compound system are shown in Table 4. The relationship between the strength of the composite gel system and the gelation time is shown in Figure 9. Under a certain mass fraction of HPAM and organic chromium cross-linking agent, and with an increased particle size of the added prefabricated gel particle plugging agent, the breakthrough vacuum degree of different compound gel systems under the same gel formation time increases. In particular, the strength of the gel compound system increases more obviously after adding WQ-100.

**Figure 10:**
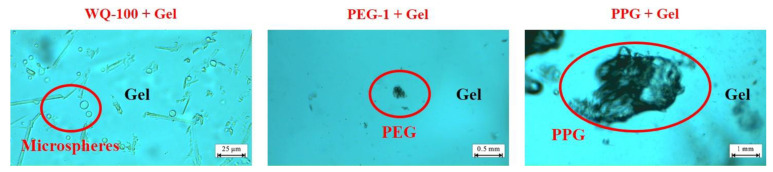
Micrograph of enhanced gels systems.

The microscopic test results of the enhanced gel systems are shown in Figure 10. The dispersion performance of the WQ-100 system gel is the best and it is evenly distributed. Compared with WQ-100, PEG-1 and PPG are both observed to have obvious granularity, indicating that the dispersion effect of PEG-1 and PPG is poor.

**Figure 11:**
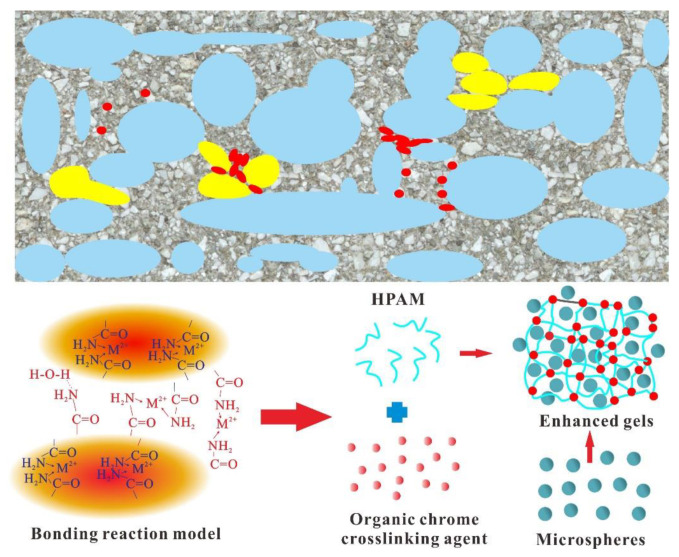
Formation mechanisms of enhanced gels with microspheres.

Generally, there are two ways to strengthen the gel. One is to add micro-nano-scale particles into the HPAM solution to strengthen the gel structure by using it as a skeleton. The second is that the HPAM solution and the added prefabricated gel particles strengthen the gel by forming additional chemical bonds. Figure 11 shows the mechanism of action of nanosphere-enhanced gel and PEG/PPG particle-enhanced gel. The red and yellow areas represent small particle plugging agents such as microspheres and large particle plugging agents such as PEG and PPG, respectively. For the micro-nano-enhanced bulk gel system, if nano-SiO_2_ particles are added, it does not participate in the coordination reaction due to their stable chemical properties; it only exists in the form of a gel skeleton [23]. The essence of nano-microspheres is AM and its derivatives with a certain stable structure, so it can not only serve as the skeleton of the gel during the formation of the gel. In addition, the nano-microspheres can form coordination bonds with the colloidal HPAM dispersion system through the cross-linking agent, which leads to the largest improvement in the breakthrough vacuum degree of the gel system with the addition of microspheres [24].

The structural properties and particle size of PEG enable it to form a reinforced structure in part of the gel. In contrast, the PPG particles can develop coordination with the HPAM dispersion system on its surface, which can both improve the gel strength to a certain extent. However, due to the poor dispersibility of PEG and PPG in the liquid phase, the gel can only be strengthened locally. Meanwhile, the larger particle size of PEG and PPG particles also aggravated the injection difficulty of the gel system.

3. Enhanced foam with pre-crosslinking gel particle and gels

**Figure 12:**
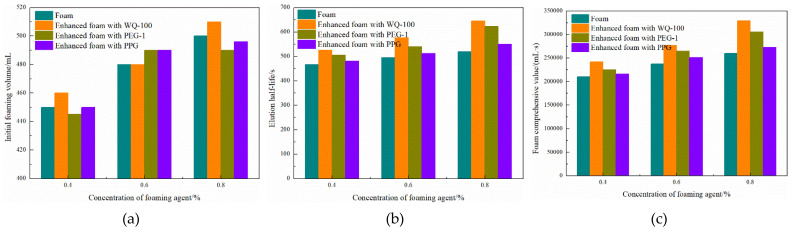
The basic performance of enhanced foam system and single foam: (**a**) foam volume, (**b**) foam liquid chromatography half-life, (**c**) foam composite value.

The foaming performance of the reinforced foam system is shown in Figure 12 on the right. The foaming volume of microsphere-reinforced foam is more significant than that of single foam. The foaming volume of the reinforced foam system obtained by compounding other prefabricated particle plugging agents except microspheres is smaller than that of single foam. Combined with the results of the liquid elution half-life test, the foaming agent has good tolerance to the actual working environment with a small amount of white oil in the solution. However, since the specific surface area of PEG particles is larger than that of PPG, more white oil is attached to the surface of the PEG particles during sampling, resulting in a more obvious attenuation of the reduction of the foaming volume and half-life.

**Figure 13:**
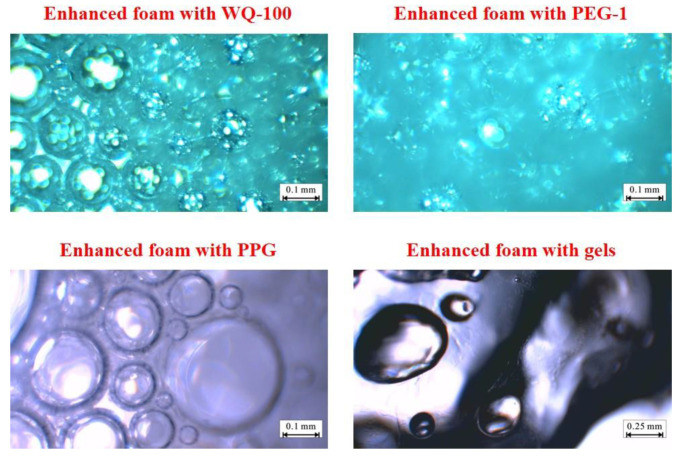
Enhanced foam system with pre-crosslinking gel particle and gels.

Sampling and microscopic observation of the reinforced foam system are carried out, and the results are shown in Figure 13. A single conventional foam liquid film is thinner, the particle size distribution of the bubble particles is relatively concentrated, and the liquid phase is clear and transparent. In the PEG and PPG reinforced foam system, it was observed that the particle size homogeneity of the bubble particles decreased significantly, no other particles were seen on the surface of the bubble particles, and the liquid phase was turbid. After PEG and PPG plugging agents were added to the foaming agent system, they did not participate in the foaming process. Only the surface white oil entered the liquid phase, resulting in the deterioration of the foaming performance of the foaming agent.

In the microsphere-reinforced foam system, it can be seen that a large number of microspheres are adsorbed on the surface of the bubble particles, the particle size of the bubble particles is concentrated, and the particle size is reduced. Since the microspheres are originally dispersed in the oil phase, the liquid phase is slightly turbid, but the weakening effect of the oil relative to the foaming agent is weak. For microsphere-reinforced foam, the microspheres are uniformly dispersed in the foaming agent solution and form a skeleton structure on the liquid film after foaming. During the foaming process, the microspheres absorb water and expand, which reduces the free water in the liquid film and strengthens the ability of the foaming agent to have interfacial tension. Additionally, the polymer surface has a large number of hydroxyl groups, negative ions appear in free water after dispersion, and the diffusion double electron layer appears, strengthening the repulsion between the liquid films and increasing the foam volume [25]. On the other hand, in the process of liquid film dehydration, the microspheres are dehydrated synchronously, resulting in the slowing down of the water loss rate of the foam on the macroscopic level, which is manifested as the prolongation of the half-life of the foam. The synergistic effect of the two mechanisms greatly increases the comprehensive value of the foam. The liquid film of the gel-reinforced foam system is thicker and there is basically no contact between the bubbles. After the foaming, the liquid phase of the gel is still the main body and the foaming effect of the foaming agent on the gel is extremely poor.

### 3.2. Plugging and EOR Performance of Gel System

#### 3.2.1. Plugging Performance

1. Porous reservoir

Since the porosity reservoir model is a low-porosity and low-permeability reservoir, only the microspheres and PEG systems with smaller particle sizes are selected for the plugging performance evaluation experiment. The plugging effects of microspheres and PEG systems on porous reservoirs with different permeability are shown in Table 5. Compared with microspheres, the PEG system has a poor plugging effect in reservoirs with low permeability but has a stronger plugging effect on reservoirs with high permeability. As the permeability increased from 4.8 × 10^−3^ μm^2^ to 156.7 × 10^−3^ μm^2^, the plugging ability of the microsphere system decreased rapidly and the plugging rate decreased from 92.05% to 20.73%. The PEG system can play a good plugging role in the reservoir with relatively high permeability; the plugging rate decreases from 96.83% to 91.82%.

**Figure 14:**
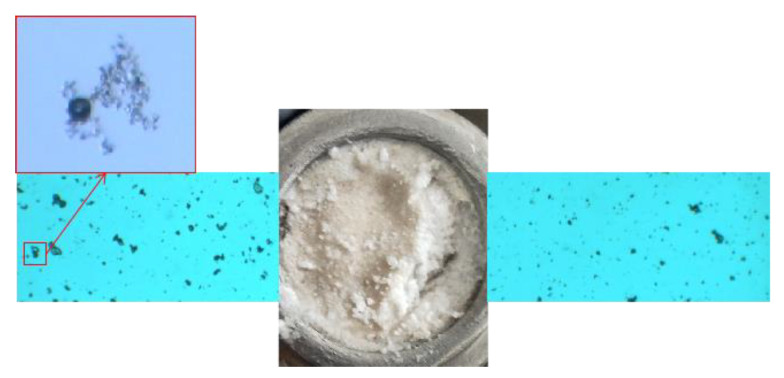
Micrograph of pore formation model’s end face and internal after injecting PEG system.

Microscopic observation was performed on the end face of the model injection end after the PEG system was pressed, and the results are shown in Figure 14. It can be observed that PEG particles accumulate on the end face of the model and fail to enter the model to play a blocking role. Since the PEG system is mainly due to the hydration expansion after injection and the plugging of the hyperpermeable pores by a single particle, it is more inclined to reduce the permeability of the hyperpermeable layer using deep regulation and flooding. In addition, the microsphere-PEG compound system (0.5%, 0.25 PV) was used to simulate the heterogeneous reservoir formed by the interlayer permeability difference through the dual sand-packing tube model, and the effect of different injection sequences on the plugging performance was studied. Taking the reservoir model with a permeability difference of 30 as an example: when the microspheres were injected first, the injection pressure only increased from 0.21 MPa to 0.24 MPa, and when the PEG system continued to be injected, the pressure increased rapidly, reaching as high as 2.48 MPa. PEG was injected first, and the pressure in the model quickly rose to 2.30 MPa. Continued injection of microspheres mainly overcomes the resistance of the PEG particles. Since PEG is displaced into the formation more profoundly by the subsequent microspheres in the system with PEG injected first, it is more difficult for the injected water to flow around. Additionally, through the process of “broken-migration-re-blocking”, the plugging of the hyperpermeable layer by PEG particles is more uniform, which further enhances the continuity of the plugging effect.

2. Fractured reservoir

Table 6 shows the evaluation results of the plugging performance of a single prefabricated gel particle plugging agent. The injection pressure of the microspheres grows from 1.88 × 10^−3^ MPa to 2.5 × 10^−3^ MPa when the fracture opening is 50 μm. However, with the further increase of the fracture opening, its plugging ability disappears. The plugging ability of the PEG system began to decline when the permeability was greater than 180 μm, and the PEG particles quickly flowed out along the fracture, resulting in the final injection pressure of only 1.3 MPa. The plugging effect of microspheres and PEG decreased rapidly with the increase in fracture opening. The plugging rate of large-sized bulk particles and gel systems increased in the range of low fracture opening (50–180 μm).

The plugging performance of the prefabricated gel particle composite plugging system is shown in Table 7. The microsphere-enhanced gel system showed stronger erosion resistance than the bulk particle-PEG system in formations with larger fracture openings. It showed a higher residual plugging rate at the end of the flooding. For large-scale fractures, the prefabricated gel particle plugging agent sheared and broken by the filler is more likely to be flushed out with the displacing fluid, thus showing insufficient plugging durability for large-scale fractures.

3. Pore-fractured reservoir

**Figure 15:**
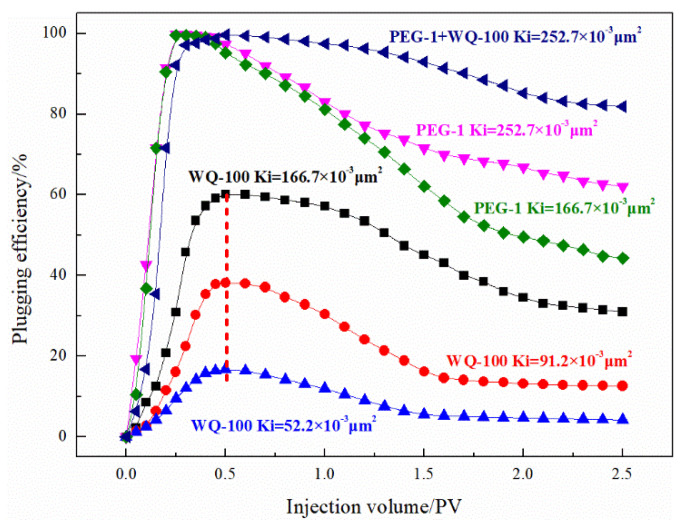
Plugging effects of pre-crosslinking gel particle on the pore-fractured reservoir.

Pore-fractured reservoirs have the characteristics of both porosity and fractured reservoirs. Large-sized plugging agents have a poor injection effect, while small-sized plugging agents are prone to channeling. Figure 15 shows the plugging effect of microspheres, PEG, and their composite systems in the pore and fractured reservoirs. The microsphere-PEG compound system showed an excellent plugging effect on pore fractured reservoirs because the compound system appeared in the 52.2 × 10^−3^ μm^2^, 91.2 × 10^−3^ μm^2^, and 166.7 × 10^−3^ μm^2^ models. The end face is blocked and only the experimental results are obtained for the reservoir with a permeability of 252.7 × 10^−3^ μm^2^. The plugging effect of the granular system on pore and fractured reservoirs is unstable, and a reinforced foam system should be developed for plugging.

#### 3.2.2. EOR Performance

For different heterogeneous reservoir models, the enhanced oil recovery effect of each gel plugging system is shown in Table 8. Compared with the 2-segment plugging system, the recovery degree of the 4-segment plugging system after the first round of PEG-microsphere injection was basically the same as that after the 2-segment plugging system was injected with PEG particles. In the first stage, the front-end PEG particles are mainly used to block and increase the swept area. After injecting the second round of PEG-microspheres, a new slug appeared in the 4-segment plugging system, which additionally blocked the inlet section of the high-permeability pipe and further forced the injected fluid to enter the low-permeability pipe for oil displacement. The level of output increased significantly. Subsequent microsphere systems are also mainly turned into low-permeability tubes, relying on the deformation and adsorption capacity of the microspheres to the oil phase to further utilize the residual oil. After plugging, the recovery factors of the three models were increased by 11.49%, 24.14%, and 25.89%, respectively. Increasing the number of slugs had a positive effect on the control and flooding performance. Compared with heterogeneous porosity reservoirs, the recovery degree of fractured reservoirs before plugging is low, only about 16%.

**Figure 16:**
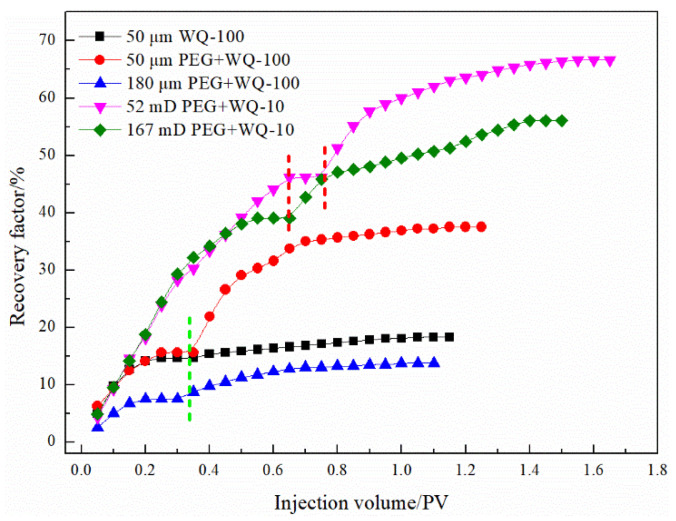
EOR effects of enhanced gels on fractured and pore-fractured reservoir.

As shown in Figure 16, take the crack width of 50 μm and the PEG-microsphere system as an example; after all the PEG particles were injected, the recovery rate reached 31.56%. The injection of microspheres and the adsorption of PEG particles further blocked the hypertonic channel. In addition, the microspheres entered the low-permeability matrix together with the water phase and elastically deformed in the matrix pores, further producing the residual oil that was not used during the water flooding process. Continued water injection replaced the pre-microsphere slug in the low-permeability reservoir. Because the length of the slug formed by PEG particles failed to seal the entire fracture, the final recovery rate reached 37.50%. For the two reservoir models, the PEG-microsphere system enhanced oil recovery by 21.87% and 6.25%, respectively, and the PPG-PEG system enhanced oil recovery by 27.77% and 21.95%, respectively. The bulky particles have a good ability to control water and profile in fractured reservoirs, and the microsphere system also has a stronger oil washing effect than the single water phase after turning to the matrix.

For pore-fractured reservoirs, after the microsphere system is injected into a reservoir with high fracture density, due to the extremely small particle size and good fluidity, the amount of microspheres per unit fracture area decreases and the bridging instability between microsphere particles intensifies. The injected fluid can only perform secondary oil washing near the wall of the micro-cracks, so it enters the failure stage immediately after stopping the injection of microspheres, and the PEG-microsphere system can form a more reliable plugging. The microsphere system improves the oil recovery of porous and fractured reservoirs with permeability by 8.75% and 3.95%, respectively, and the PEG-microsphere composite system improves the oil recovery by 20.52% and 17.08%, respectively, as shown in Figure 16. The addition of the PEG front slug effectively blocked the micro-fractures, and the subsequent microsphere system formed adsorption on the PEG surface and further strengthened the diversion of the injected water to the formation.

### 3.3. Plugging and EOR Performance of Gel Enhanced Foam System

#### 3.3.1. Plugging Performance

The plugging effects of the enhanced foam system on different reservoirs are shown in Table 9. Compared with the conventional foam system, the microsphere foam system has a higher comprehensive foam value, which means that its blocking performance is stronger than that of the conventional foam system in static experiments. In the microsphere foam system, after the foam fails, the microspheres—as foam stabilizers—continue to play a blocking role. Compared with the microsphere-enhanced foam system, the “break-migration-re-plugging” process of the PEG-microsphere system in the porous reservoir significantly slows down the decay rate of the plugging rate. After the foam system is injected into the fracture, it quickly flows back along the fracture, which is not suitable for formations with a fractured opening greater than 50 μm. Compared with the conventional jelly system, the structure of foam-modified jelly is more complex, and it is more difficult to break through the foam-modified jelly under the same injection amount. Microsphere-enhanced foam can be used for secondary blocking through microspheres to enhance the blocking ability.

#### 3.3.2. EOR Performance

**Figure 17:**
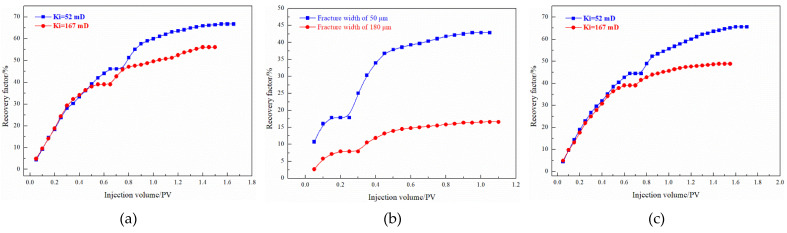
EOR effects of enhanced foam with WQ-100: (**a**) pore reservoirs, (**b**) fracture reservoirs, (**c**) pore-fractured reservoir.

The enhanced oil recovery effect of the enhanced foam system on different heterogeneous reservoir models are shown in Table 10 and Figure 17. Compared with the 4 slug PEG-microsphere plugging system, the conventional foam and the microsphere-enhanced foam system can increase the recovery by 0.20% and 2.16%, respectively. Compared with the PEG-microsphere system, the foam system has a specific surfactant flooding ability while plugging, and the recovery degree is improved more greatly. For microsphere-enhanced foam, effective plugging was not formed in fractured formations with a fractured opening of 180 μm, only foaming agents and microspheres could be used to strengthen oil washing near the fracture surface, and a large amount of injected fluid was lost. The gel-reinforced foam system shows good plugging and profile control performance. Forming a gel system with worse rheology strengthens the ability to divert the injected water, allows the subsequent fluid to enter the depth of the matrix for oil washing, and increases the reserves in the low-permeability matrix [26]. After injection of the gel-enhanced foam system, the recovery factors of fractured reservoirs with fracture widths of 50 and 80 μm increased by 29.41% and 24.39%, respectively. The increase in fracture density leads to an aggravation of the ineffective circulation of injected fluid, which requires higher plugging, regulation, and displacement capability of the same plugging system. Compared with the PEG-microsphere composite system, the recovery degree of the microsphere-enhanced foam increased by 3.92% and 4.35% under the reservoir conditions of 52 mD and 167 mD.

### 3.4. Construction of Profile and Control System Technique Plate

**Figure 18:**
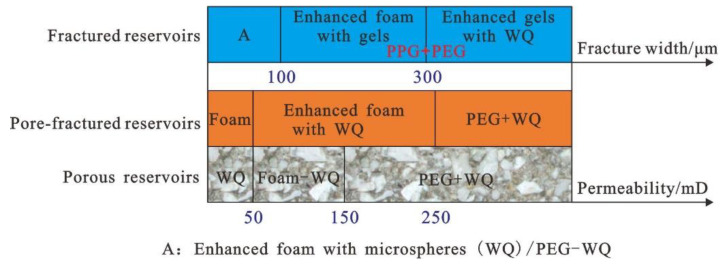
Technique boundary chart of plugging and flooding system in heterogeneous reservoirs.

Combined with the static performance, plugging mechanism, and enhanced oil recovery effect of the reinforced gel and reinforced foam systems, a technical chart of the plugging system was constructed according to the characteristics of different heterogeneous reservoirs, as shown in Figure 18. For pore-fractured reservoirs with a permeability of 50–200 mD, considering the stability of injection, the microsphere-reinforced foam system is still preferred to strengthen the plugging of micro-fractures and play the role of micro-spheres in regulating plugging. For pore-fractured reservoirs with a permeability higher than 200 mD, the reservoirs have begun to show obvious fractured reservoir characteristics, so the PEG-microsphere system with good injection performance should be prioritized to plug microfractures. For fractured reservoirs with fracture width less than 100 μm (permeability is about 400,000 × 10^−3^ μm^2^ according to the empirical formula, which is affected by porosity in fractures), considering the effectiveness of plugging and reducing injection resistance, the use of microspheres-reinforced foam system or PEG-microsphere system. Considering the stronger mechanical strength of microsphere-enhanced gel, microsphere-enhanced gel can be used in fractured reservoirs with a fractured opening of more than 300 μm to enhance the plugging effect. Alternatively, the formation of bulk swelling particles and PEG particles can comprehensively strengthen the plugging of fractures and improve the fluid flow diversion ability, followed by the use of microsphere flooding to improve the oil washing efficiency of the remaining oil in the low-permeability matrix and comprehensively improve the recovery factor.

## 4. Conclusions

The conclusions of this work are as follows.

(1) The adsorption effect of microspheres (WQ-100) on the surface of PEG-1 is more potent than that of PPG, and the deposition is mainly on the surface of PPG. The adsorption effect of PEG-1 on the surface of PPG is not apparent, primarily manifested as deposition stacking.
(2) The gel was synthesized with 0.2% HPAM + 0.2% organic chromium cross-linking agent, and the strength of enhanced gel with WQ-100 was higher than that of PEG-1 and PPG. The comprehensive value of WQ-100 reinforced foam is greater than that of PEG-1, and PPG reinforced foam and the enhanced foam with gel has a thick liquid film and poor foaming effect.
(3) For the heterogeneous porous reservoir with the permeability of 5/100 mD, the enhanced foam with WQ-100 shows better performance in plugging control and flooding, and the recovery factor increases by 28.05%. The improved foam with gel enhances the fluid flow diversion ability and the recovery factor of fractured reservoirs with fracture widths of 50 μm and 180 μm increases by 29.41% and 24.39%.
(4) For pore-fractured reservoirs with a permeability of 52/167 mD, the PEG + WQ-100 microsphere and enhanced foam with WQ-100 systems show better plugging and recovering performance, and the recovery factor increases are 20.52% and 17.08%, 24.44% and 21.43%, respectively.
(5) The plugging performance of the composite gel system is stronger than that of the enhanced gel with foam. However, the oil displacement performance of the gel-enhanced foam is better than that of the composite gel system due to the “plug-flooding-integrated” feature of the foam.

## Figures and Tables

**Table 1:** The formula of enhanced foam system with pre-crosslinking gel particles.

Enhanced Foam System	Interfacial Tension/(mN/m)	Note
0.4% foaming agent	35.2	/
0.4% foaming agent + 0.1% WQ-100	33.6	Reduce surface tension
0.4% foaming agent + 0.1% PEG-1	34.8	Not significantly
0.4% foaming agent + 0.1% PPG	/	Particle size is too large to measure
0.4% foaming agent + 0.2% HPAM + 0.1% organic chromium cross-linking agent	46.5	Liquid film gelation, foam stabilization

**Table 2:** Basic parameters of the fractured and pore-fractured experimental models.

Physical Models	Fracture Width /μm	Porosity before Splitting /%	Permeability before Splitting /× 10^−3^ μm^2^	Permeability after Splitting /× 10^−3^ μm^2^
Fractured models	50	19.5	5.3	90,000
	180	20.6	4.6	2,480,000
	340	21.2	6	31,200,000
	510	20.4	4.7	87,000,000
Pore-fractured models	Matrix volume /cm^3^	Microfracture volume/cm^3^	Porosity /%	Permeability /× 10^−3^ μm^2^
	295	0.4	27.1	52.2
		0.8	28.4	91.2
		1.2	29.8	166.7
		1.6	31.2	252.7

**Table 3:** Foam comprehensive coefficient under varying concentrations of foaming agent.

Concentration of Foaming Agent/%	Initial Foaming Volume/mL	Elution Half-Life/s	Foam Composite Value/(mL s)
0.1	160	83	13,280
0.2	230	262	60,260
0.4	450	467	210,150
0.6	480	495	237,600
0.8	500	520	260,000
1.0	520	515	267,800

**Table 4:** Strength and gelation time of gel compounds system.

HPAM/%	Gel Agent	Organic Chromium Crosslinking Agent/%	Breakthrough Vacuum /MPa	Gelation Time/h
0.2		0.1	0.024	21
		0.2	0.027	12
		0.3	0.031	7
	0.2% WQ-100	0.1	0.038	21.5
		0.2	0.040	12
		0.3	0.042	7.5
	0.2% PEG-1	0.1	0.032	22
		0.2	0.035	12
		0.3	0.037	8
	0.2% PPG	0.1	0.028	22
		0.2	0.032	13
		0.3	0.033	7

**Table 5:** Plugging efficiency of pre-crosslinking gel particle on the porous reservoir.

Plugging System	Initial Permeability /10^−3^ μm^2^	Plugging Efficiency /%	Plugging Efficiency after Injection 10 PV Formation Water
0.5% WQ-100 0.5 PV	4.8	92.05	79.21
	15.9	90.52	71.93
	49.6	78.21	56.20
	99.8	52.34	21.54
	156.7	20.73	5.80
0.5% PEG-1 0.5 PV	4.8	End face seal	
	15.9	End face seal	
	49.6	96.83	90.24
	99.8	93.83	82.23
	156.7	91.82	77.53

**Table 6:** Plugging efficiency of pre-crosslinking gel particle on the fractured reservoir.

Plugging System	Fracture Width /μm	Plugging Efficiency /%	Residual Plugging Efficiency/%
0.5% WQ-100 (0.5 PV)	50	24.80	7.39
	180	16.67	5.17
	340	5.00	0.00
	510	1.23	0.00
0.5% PEG-1 (0.5 PV)	50	94.29	86.88
	180	82.98	38.46
	340	18.18	6.90
	510	13.64	0.72
0.5% PPG (0.5 PV)	50	98.76	91.83
	180	99.76	99.34
	340	99.53	98.49
	510	99.68	98.75
0.2% HPAM + 0.2% Cr^3+^ (0.5 PV)	50	97.11	90.91
	180	99.81	99.18
	340	99.88	99.67
	510	99.91	99.75

**Table 7:** Plugging efficiency of multiple pre-crosslinking gel particles on the fractured reservoir.

Plugging System	Fracture Width /μm	Plugging Efficiency /%	Residual Plugging Efficiency/%
0.5% PPG 0.25 PV + 0.5% PEG-1 0.25 PV	50	99.02	97.61
	180	99.85	99.50
	340	99.87	99.66
	510	99.90	99.81
0.5% WQ-100 + 0.2% HPAM + 0.2% Cr^3+^ 0.5 PV	50	98.93	92.85
	180	99.91	98.97
	340	99.90	99.05
	510	99.92	99.13

**Table 8:** Plugging efficiency of multiple pre-crosslinking gel particle on the fractured reservoir.

Reservoir Models		Plugging System Formula	EOR before Plugging /%	EOR after Plugging /%
Porous reservoirs	50/100 mD	0.5 PV WQ-100	39.19	50.68
		0.25 PV PEG + 0.25 PV WQ-100	38.82	64.71
		0.15 PV PEG + 0.15 PV WQ-100 + 0.1 PV PEG + 0.1 PV WQ-100	39.08	63.22
Fractured reservoirs	Fracture width /μm	Plugging system Formula	EOR before plugging /%	EOR after plugging /%
	50	0.5 PV WQ-100	14.63	18.29
	50	0.2 PV PEG + 0.3 PV WQ-100	15.63	37.5
	180		7.5	13.75
	50	0.2 PV PPG + 0.3 PV PEG	16.67	44.44
	180		7.32	29.27
Pore-fractured reservoirs	Permeability /mD	Plugging system Formula	EOR before plugging /%	EOR after plugging /%
	52	0.5 PV WQ-100	43.75	52.5
	167		40.79	44.74
	52	0.2 PV PEG + 0.3 PV WQ-100	46.15	66.67
	167		39.02	56.1

**Table 9:** Plugging efficiency of enhanced foam system in varying reservoir models.

Reservoirs Models	Permeability/mD	0.25 PV PEG + 0.25 PV WQ-100	0.25 PV Foam + 0.25 PV WQ-100
Porous reservoirs	5/150	Section block	94.2%/97.8%
		96.1%/97.6%	94.1%/96.3%
		92.8%/94.5%	91.6%/92.5%
		91.1%/92.8%	90.4%/90.9%
Reservoirs models	Fracture width/μm	Foam + WQ-100	Enhanced foam with gels + WQ-100
Fractured reservoirs	50	85.5/90.5	94.7/96.4
	180	50.6/56.8	92.9/94.2
	340	10.3/13.8	91.6/92.6
	510	4.8/5.9	89.7/90.5
Reservoirs models	Microfracture/matrix volume ratio	Foam	WQ-100 + Foam
Pore-fractured reservoirs	0.4/295	91.4	93.6
	0.8/295	89.7	92.8
	1.2/295	86.1	91.2
	1.6/295	80.3	90.5

**Table 10:** EOR efficiency of enhanced foam at varying reservoirs models.

Reservoir Models		Plugging System Formula	EOR before Plugging /%	EOR after Plugging /%
Porous reservoirs	5/100 mD	PEG-WQ-100	39.08	63.22
		Foam	39.13	65.22
		Enhanced foam with WQ-100	39.02	67.07
Fractured reservoirs	Fracture width/μm	Plugging system Formula	EOR before plugging /%	EOR after plugging /%
	50	PEG-WQ-100	15.63	37.5
		PPG-PEG-1	16.67	44.44
		WQ-100	14.63	18.29
		Enhanced foam with WQ-100	17.86	42.86
		Enhanced foam with gels	17.65	47.06
	180	PEG-WQ-100	7.5	13.75
		PPG-PEG-1	7.32	29.27
		Enhanced foam with WQ-100	7.89	16.58
		Enhanced foam with gels	8.54	32.93
Pore-fractured reservoirs	Permeability /mD	Plugging system Formula	EOR before plugging /%	EOR after plugging /%
	52	PEG-WQ-100	46.15	66.67
		Foam	44.44	65.56
		Enhanced foam with WQ-100	46.67	71.11
	167	PEG-WQ-100	39.02	56.1
		Foam	39.02	48.78
		Enhanced foam with WQ-100	42.86	64.29

## Data Availability

Not applicable.
